# Independent evolution for sex determination and differentiation in the *DMRT* family in animals

**DOI:** 10.1242/bio.041962

**Published:** 2019-08-15

**Authors:** Shuuji Mawaribuchi, Yuzuru Ito, Michihiko Ito

**Affiliations:** 1 Biotechnology Research Institute for Drug Discovery, National Institute of AIST, Central 5, 1-1-1 Higashi, Tsukuba, 305-8565, Japan; 2 Department of Biosciences, School of Science, Kitasato University, Kitasato 1-15-1, Minamiku, Sagamihara 252-0373, Japan

**Keywords:** *DMRT*, DM domain, Sex determination, Sex differentiation, Gene duplication

## Abstract

Some *DMRT* family genes including arthropod *dsx*, nematode *mab-3*, and vertebrate *dmrt1* are involved in sex determination and/or differentiation in bilaterian animals. Although there have been some reports about evolutionary analyses of the family by using its phylogenetic trees, it is still undecided as to whether these three sex determination-related genes share orthologous relationships or not. To clarify this question, we analyzed evolutional relationships among the family members in various bilaterians by using not only phylogenetic tree analysis, but also synteny analysis. We found that only four genes, *dmrt2a/2b*, *dmrt3*, *dmrt4/5* and *dmrt93B* were commonly present in invertebrate bilateria. The syntenies of *dmrt2a/2b*-*dmrt3* and *dmrt4/5*-*dmrt93B* are conserved before and after two rounds of whole genome duplication in the ancestral vertebrate. Importantly, this indicates that *dmrt1* must have appeared in the common vertebrate ancestor. In addition, *dmrt1*, *dsx*, or *mab-3* formed each different cluster at a distance in our phylogenetic tree. From these findings, we concluded that the three sex determination-related genes, *dmrt1*, *dsx*, and *mab-3* have no orthologous relationships, and suggested independent evolution for sex determination and differentiation in the *DMRT* gene family. Our results may supply clues about why sex-determining systems have diverged during animal evolution.

## Abstract (precis)

**Summary:** Three *DMRT* family genes, vertebrate *dmrt1*, arthropod *dsx* and nematode *mab-3*, involved in sex determination and primary sex differentiation have no orthologous relationships, indicating independent evolution in bilaterian animals.

## INTRODUCTION

The doublesex and mab-3 related transcription factor (DMRT) family is well-conserved in bilaterian animals and is characterized by a DNA-binding region called the DM domain (Matson and Zarkower, 2012). The domain was named from *Drosophila melanogaster* Dsx and *Caenorhabditis elegans* Mab-3 proteins, both of which play important roles in sex differentiation (Matson and Zarkower, 2012). Most animals have multiple *DMRT* genes. In mammals, there are eight *DMRT* genes, *DMRT1-DMRT8* (Veith et al., 2006; Bellefroid et al., 2013). Veith et al. previously suggested that *DMRT7* and *DMRT8* are mammalian-specific *DMRT* genes (Veith et al., 2006). Most of the DMRT proteins play roles in various developmental processes including myogenesis, somitogenesis, neurogenesis and gametogenesis (O'Day, 2010; Bellefroid et al., 2013; Zhang et al., 2014a; Yu et al., 2015).

Some *DMRT* genes are well-studied in sexual determination and differentiation in somatic cells of the gonads. *DMRT1* is a regulator of testicular formation and/or male determination in gonadal somatic cells in various vertebrate species (Yoshimoto and Ito, 2011; Zhao et al., 2015). *Dmrt1* is necessary for somatic-cell masculinization in mice (Raymond et al., 2000). In chickens, the Z-linked *dmrt1* induces male sex determination by its gene dosage (Smith et al., 2009). The *dmrt1* paralogs, the Y-linked *dmy*/*dmrt1by* in teleost fish (*Oryzias latipes*) and the W-linked *dmw* in the African clawed frog (*Xenopus laevis*) are sex-determining genes (Matsuda et al., 2002; Nanda et al., 2002; Yoshimoto et al., 2008; Mawaribuchi et al., 2012). On the other hand, in germ cells of the gonads, Dmrt1, Dmrt6, Dmrt7 and Dmrt11E are involved in spermatogenesis in some bilaterian species (Kawamata and Nishimori, 2006; Matson et al., 2010; Zhang et al., 2014a,b; Yu et al., 2015). Dmrt1 and Dmrt4 play roles in oogenesis and folliculogenesis, respectively, in female mice (Balciuniene et al., 2006; Krentz et al., 2011). Moreover, many *DMRT* genes are implicated in non-gonadal development. Dmrt2 participates in myogenesis and somitogenesis in some vertebrates (Meng et al., 1999; Seo et al., 2006; Sato et al., 2010). Dmrt3, Dmrt4, Dmrt5, Dmd-5 and Dmrt93B engage in neurogenesis in some bilaterian species (Huang et al., 2005; O'Day, 2010; Andersson et al., 2012; Saulnier et al., 2013; De Clercq et al., 2016; Oren-Suissa et al., 2016). The molecular function of DMRT8 is not yet known. Importantly, only the three types of the DMRT family genes – that is, *dmrt1* homologs, *dsx* and *mab-3* – are known to function in sex determination and/or somatic sex differentiation to date.

Some researchers suggested that *DMRT1* may be a vertebrate equivalent of *dsx*, that is, *dsx* ortholog (Ottolenghi et al., 2002; Kato et al., 2011; Clough et al., 2014). Other researchers discussed that they could not yet conclude that *dmrt1* is the mammalian ortholog of *dsx* and *mab-3* from their sequence comparisons (Raymond et al., 2000). Moreover, the phylogenetic trees of DMRT family proteins showed that two clusters consisting of dmrt1 and dsx do or do not form a sister group (Toyota et al., 2013; Wexler et al., 2014). In addition, the synteny analysis of *DMRT* family genes has not been reported in invertebrates. Collectively, it is still an undecided question as to whether these sex determination-related genes, *dmrt1*, *dsx*, and *mab-3* are orthologous or not. Interestingly, our recent report indicated that the ancestral gene of vertebrate *dmrt1* might have emerged not for sex determination but for germ-cell development (Mawaribuchi et al., 2017a), suggesting that *dmrt1* might not be a functional ortholog of *dsx* and *mab-3*.

The divergence of the *DMRT* family genes for gonadal and non-gonadal functions remains unclear. In addition, the synteny analysis of these genes in invertebrates is rarely performed. In this study, the evolutionary relationships of the *DMRT* genes in bilateria were examined by not only phylogenetic tree, but also synteny analysis. We found that four *DMRT* genes, *dmrt2a/2b*, *dmrt3*, *dmrt4/5* and *dmrt93B* were commonly present in invertebrate bilateria. The syntenies of *dmrt2a/2b*-*dmrt3* and *dmrt4/5*-*dmrt93B* are conserved before and after 2R-WGD in the ancestral vertebrate. As for the sex determination-related DMRT genes, the evolutionary analyses revealed that *dmrt1* might have appeared in the common vertebrate ancestor, and that there are independent and different clusters for *dmrt1*, *dsx* and *mab-3* in our phylogenetic tree. These results suggested that the three sex determination-related genes, *dmrt1*, *dsx* and *mab-3* might emerge independently in each taxon and obtain new functions for sex determination and/or primary sex differentiation.

## RESULTS AND DISCUSSION

### A common ancestor of bilateria must have possessed three *DMRT* family genes, *dmrt2a/2b*, *dmrt4/5* and *dmrt93B*

The syntenic relationships of the *DMRT* family genes in invertebrates have not been investigated in detail. To perform the synteny analyses of the family genes between invertebrate and vertebrate bilateria, we obtained the sequences from the GenBank or various genomes by BLAST search (Table S1). In mammals, there are eight *DMRT* genes, *DMRT1-DMRT8* (Veith et al., 2006; Bellefroid et al., 2013). Johnsen and Andersen reported that *dmrt2* (*dmrt2a*) and *dmrt2b* or *dmrt4* and *dmrt5* might emerge from their ancestral genes, respectively, through the two rounds of whole genome duplication (2R-WGD) (Johnsen and Andersen, 2012). This supports the finding that Dmrt4 and Dmrt5 may possess redundant roles during neurogenesis (Parlier et al., 2013). Interestingly, we could obtain no *dmrt2b* sequences in tetrapod genome databases (Table S1). In contrast, Kato et al. indicated the close relationship between *dmrt11E* and *dmrt2* or *dmrt99B* and *dmrt4/5* from their sequence similarity (Kato et al., 2008). Based on these findings, the *DMRT* gene families could be classified into eight major subsets, *DMRT1*, *DMRT2a/2b* (*dmrt2*, *dmrt2a* and *dmrt2b*), *DMRT3*, *DMRT4/5* (*dmrt4*, *dmrt5* and *dmrt99B)*, *DMRT6*, *DMRT7*, *DMRT8* and *DMRT93B*. The *dmrt93B* orthologs belonging to the eighth *DMRT* family were newly identified in not only invertebrates, but also some vertebrate species (Table S1, see details below). Importantly, the BLAST search revealed that only four genes, *dmrt2a/2b*, *dmrt3*, *dmrt4/5* and *dmrt93B* were commonly present in invertebrate bilateria (Table S1).

**Fig. 1.**
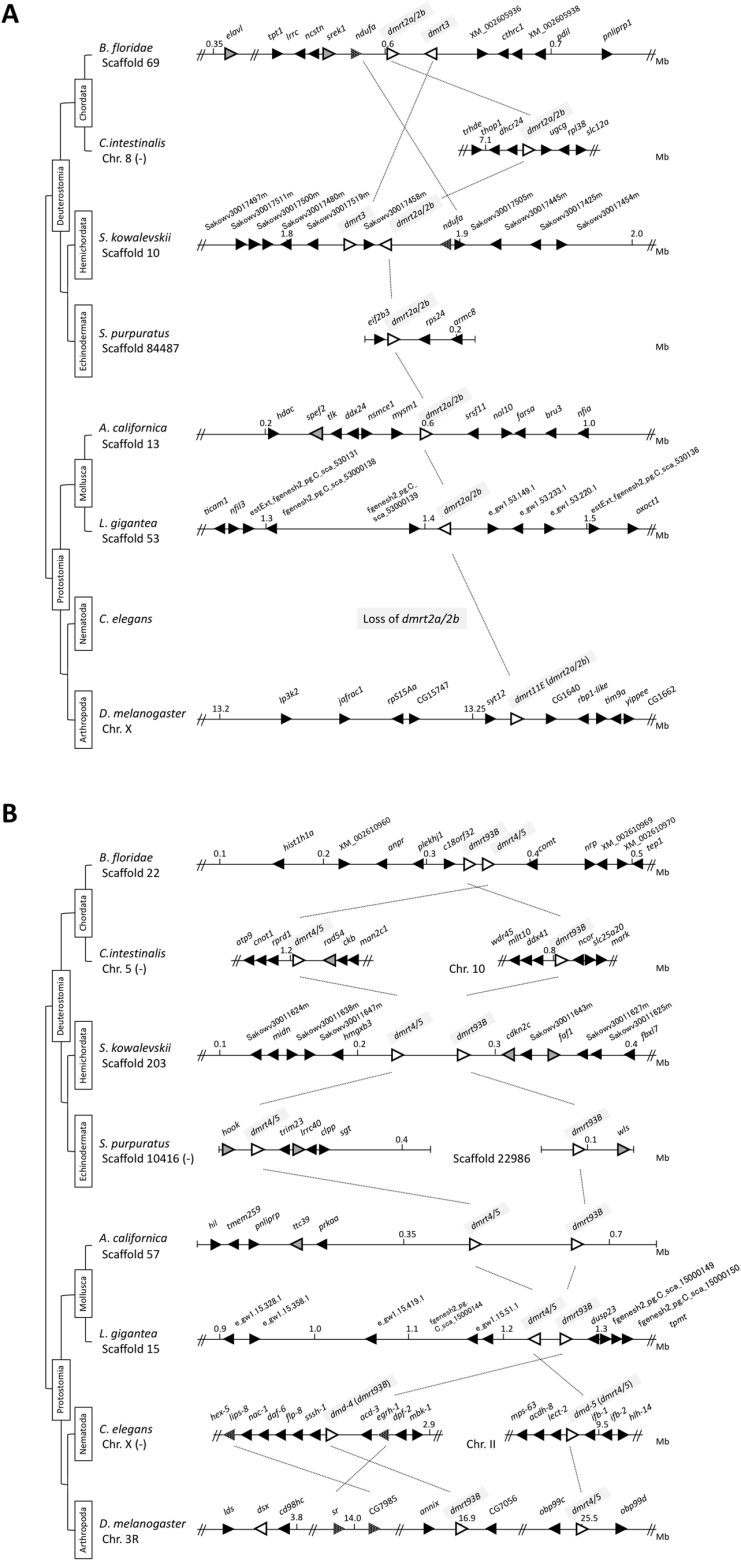
**Synteny analysis of *DMRT* genes in invertebrates.** (A) Synteny of *dmrt2a/b* (*dmrt11E*) and *dmrt3*. (B) Synteny of *dmrt4/5* (*dmrt99B*) and *dmrt93B*. The synteny analysis was performed in eight species of invertebrate bilateria (*B.* *floridae*; *C.* *intestinalis*; *S.* *kowalevskii*; *S.* *purpuratus*; *A.* *californica*; *L.* *gigantea*; *C.* *elegans*; *D.* *melanogaster*). Triangles indicate genes and their tips correspond to their 3′-ends. White and black triangles represent *DMRT* genes and surrounding genes, respectively. Gray triangles represent genes that have been found in the areas surrounding *DMRT* genes in both vertebrates and invertebrates (Fig. 2). Spotted triangles represent genes showing synteny between invertebrates. Chr., chromosome; (−), reverse relationship.

Then we performed synteny analyses of the *DMRT* genes in invertebrate bilateria including four deuterostome species (Chordata, Cephalochordata, *Branchiostoma floridae*; Chordata, Urochordata, *Ciona intestinalis*; Hemichordata, *Saccoglossus kowalevskii*; Echinodermata, *Strongylocentrotus purpuratus*) and four protostome species (Mollusca, *Aplysia californica*; Mollusca, *Lottia gigantea*; Nematoda, *C.* *elegans*; Arthropoda, *D.* *melanogaster*) (Fig. 1). A synteny analysis of *dmrt2a/2b* in these eight species indicated the presence of the *dmrt3* gene in close proximity to the *dmrt2a/2b* locus in deuterostomia (Fig. 1A). In deuterostomia, three genes, *dmrt2a/2b*, *dmrt3* and *ndufa* were found to be syntenic between *B. floridae* and *S. kowalevskii*, suggesting that *dmrt3* could have emerged through a gene duplication event of *dmrt2a/2b* during deuterostome evolution (Fig. 1A). In addition, we found that the *dmrt4/5* (*dmrt99B*) and *dmrt93B* genes were located adjacent to each other in two deuterostomes (*B. floridae* and *S. kowalevskii*) and two protostomes (*A. californica* and *L. gigantea*) (Fig. 1B). These two genes were present on the same chromosome in *D*. *melanogaster* (Fig. 1B). CG7985 (*lips-8*) and *sr* (*egrh-1*) appeared to be located near *dmrt93B* (*dmd-4*) in *D*. *melanogaster* and *C. elegans* (Fig. 1B). Taken together, these results suggested that a common ancestor before the divergence of deuterostomes and protostomes possessed three subsets of the *DMRT* gene family, *dmrt2a/2b*, *dmrt4/5* and *dmrt93B*.

**Fig. 2.**
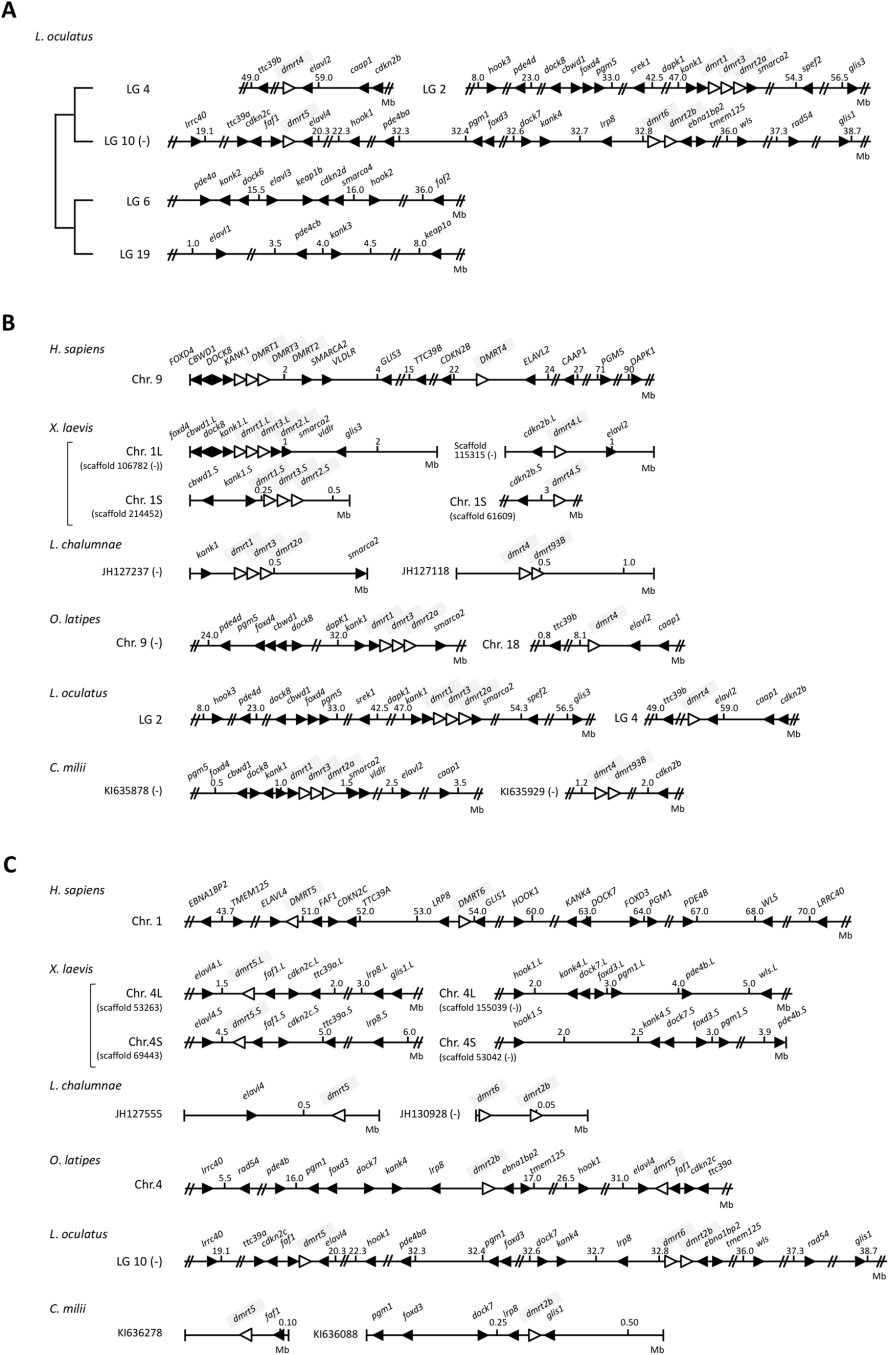
**Synteny analysis of *DMRT* genes in vertebrates.** (A) Synteny of *DMRT* family genes in *L.* *oculatus*. This synteny shows a trace of 2R-WGD. (B) Synteny of *DMRT1*, *DMRT2*, *DMRT3*, *DMRT4* and *dmrt93B*. (C) Synteny of *dmrt2b*, *DMRT5* and *DMRT6*. The synteny analyses were performed in six species of vertebrates (*H.* *sapiens*, *X.* *laevis*, *L.* *chalumnae, O. latipes*, *L.* *oculatus* and *C.* *milii*). Triangles indicate genes and their tips correspond to their 3′-ends. White and black triangles represent *DMRT* genes and the surrounding genes, respectively. Chr., chromosome; LG, linkage group; (−), reverse relationship.

### The syntenies of *dmrt2a/2b*-*dmrt3* and *dmrt4/5*-*dmrt93B* are conserved before and after two rounds of whole genome duplication (2R-WGD) in a common ancestor of vertebrates

From the above results, we found that *dmrt2a/2b*, *dmrt3*, *dmrt4/5* and *dmrt93B* might be present in a common vertebrate ancestor. It is believed that 2R-WGD occurred in the common ancestor of vertebrates. Based on this premise, we next examined synteny relationships of the *DMRT* family genes between invertebrates and vertebrates (Fig. 2). In the spotted gar *Lepisosteus oculatus*, which belongs to the Holostei infraclass in the Actinopterygii class, the two *dmrt* clusters *dmrt1*-*dmrt3*-*dmrt2a* and *dmrt6*-*dmr2b* were localized to the region encompassing the *hook*, *pde4*, *dock*, *foxd*, *pgm*, *kank* and *glis* family members in linkage groups (LG) 2 and 10, respectively (Fig. 2A). In addition, *dmrt4* or *dmrt5* was localized to the region encompassing the *ttc39*, *elavl* and *cdkn2* family members in LG4 or LG10, respectively. Two sets of *faf*, *foxd*, *glis*, *keap1*, *smarca* and *pgm*, three sets of *cdkn2*, *dock* and *hook*, or four sets of *elavl*, *kank* and *pde* paralogues were observed in the linkage groups in close proximity to the *dmrt* family members, indicating the presence of traces of 2R-WGD (Fig. 2A). A gene corresponding to the ancestral gene of the *elavl* family was localized in the vicinity of *dmrt2a/2b* and *dmrt3* on scaffold 69 in *B. floridae* (Fig. 1A). The ancestral *cdkn2*- and *faf*-related genes or ancestral *ttc39*-related gene were found near *dmrt4/5* or *dmrt93B* on scaffold 203 in *S. kowalevskii* and scaffold 57 in *A. californica*, respectively (Fig. 1B). The *hook*-related gene was found in the vicinity of *dmrt4/5* on scaffold 10416 in *S. purpuratus*. Moreover, *srek1* on scaffold 69 and *spef2* on scaffold 13 in *B. floridae* and *A. californica*, respectively, near *dmrt2a/2b* corresponded to the regions around *dmrt1*-*dmrt3*-*dmrt2a* cluster on LG 2 in *L. oculatus* (Figs 1A and 2A). *rad54* near *dmrt4/5* on chromosome 5 in *C. intestinalis*, *lrrc40* near *dmrt4/5* on scaffold 10416 in *S. purpuratus*, and *wls* near *dmrt93B* on scaffold 22986 in *S. purpuratus* corresponded to the regions on LG 10 in *L. oculatus* (Figs 1B and 2A). Interestingly, no *dmrt* genes were identified on LG6 or LG19 (Fig. 2A).

We next performed synteny analysis of *DMRT* family genes using six species of vertebrates, mammalian *Homo sapiens*, amphibian *X.* *laevis*, sarcopterygian *Latimeria chalumnae*, actinopterygian *O.* *latipes* and *L. oculatus*, and chondrichthyan *Callorhinchus milii* (Fig. 2B,C). The synteny of the *DMRT1*-*DMRT3*-*DMRT2* cluster was well-conserved in all of the vertebrate species examined (Fig. 2B). However, the *dmrt6*-*dmr2b* cluster was only conserved in the spotted gar (*L. oculatus*) and coelacanth (*L. chalumnae*) (Fig. 2C). The tandem arrays of *dmrt4* and *dmrt93B* were conserved in the elephant shark (*C. milii*) and coelacanth (*L. chalumnae*) (Fig. 2B) and in some invertebrate species (Fig. 1B). Namely, the syntenies of *dmrt2a/2b*-*dmrt3* and *dmrt4/5*-*dmrt93B* are conserved before and after the 2R-WGD. *dmrt2a* and *dmrt2b* or *dmrt4* and *dmrt5* must have evolved from *dmrt2a/2b* or *dmrt4/5*, respectively, through the 2R-WGD.

### *DMRT1* and *DMRT6* are vertebrate-specific genes

**Fig. 3.**
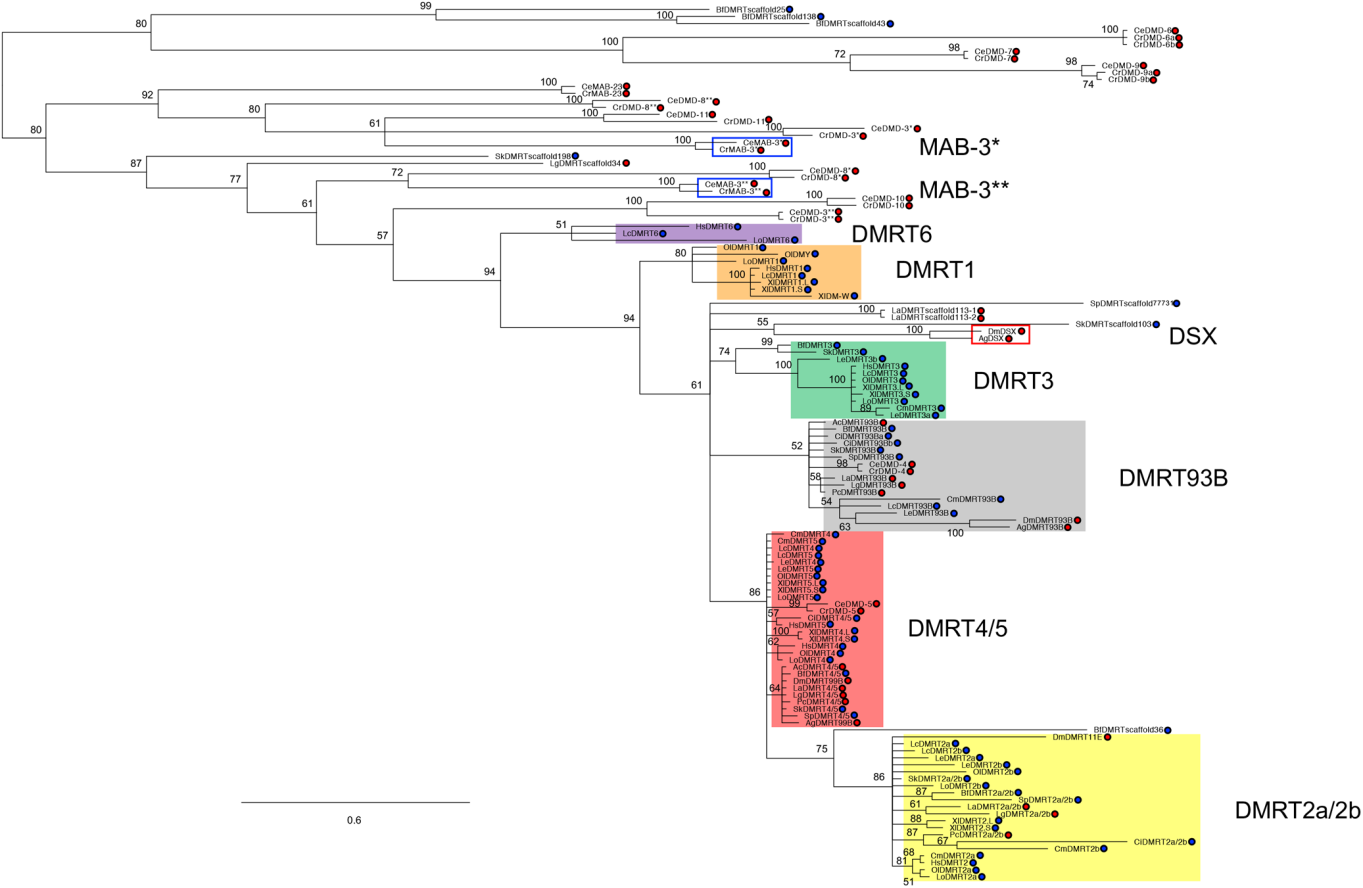
**Bayesian tree of bilaterian *DMRT* family genes.** The tree was constructed by MrBayes5D using the protein sequences of the DM domains from 19 species representing eight different phyla in bilateria (see Fig. S3). Brachiopoda, *Lingula anatina* (La); Mollusca, *Aplysia californica* (Ac); Mollusca, *L.* *gigantea* (Lg); Priapulida, *Priapulus caudatus* (Pc); Nematoda, *C.* *elegans* (Ce); Nematoda, *Caenorhabditis remanei* (Cr); Arthropoda, *Anopheles gambiae* (Ag); Arthropoda, *D.* *melanogaster* (Dm); Hemichordata, *Saccoglossus kowalevskii* (Sk); Echinodermata, *Strongylocentrotus purpuratus* (Sp); Chordata, Urochordata, *Ciona intestinalis* (Ci); Chordata, Cephalochordata, *Branchiostoma floridae* (Bf); Chordata, Vertebrata, Chondrichthyes, *C.* *milii* (Cm); Chordata, Vertebrata, Chondrichthyes, *L.* *erinacea* (Le); Chordata, Vertebrata, Actinopterygii, *L.* *oculatus* (Lo); Chordata, Vertebrata, Actinopterygii, *O.* *latipes* (Ol); Chordata, Vertebrata, Sarcopterygii, *L.* *chalumnae* (Lc); Chordata, Vertebrata, Amphibia, *X.* *laevis* (Xl); Chordata, Vertebrata, Mammalia, *H.* *sapiens* (Hs). Model test was performed by Aminosan (rtREV+F_Gamma). Blue and red circles represent Deuterostomia and Protostomia, respectively. * and ** indicate DM domain regions on 5′ and 3′ sides, respectively. The numbers indicate posterior probability. The values less than 50% were collapsed.

*DMRT2a/2b*, *DMRT3*, *DMRT4/5* and *DMRT93B* were commonly present in invertebrate bilateria (Figs 1, 2; Table S1). The synteny analyses also indicated that *DMRT7* or *DMRT8* are specific in mammalian and reptilian or mammalian species, respectively (Fig. S1). Our recent study reported that lamprey *dmrt1* is primarily expressed in germ cells, suggesting that the ancestral vertebrate *dmrt1* might have emerged for germ-cell development (Mawaribuchi et al., 2017a). We also found that *dmrt6* was pseudogenized in chondrichthyes, *Leucoraja erinacea* (Table S1). To clarify when *dmrt1* and *dmrt6* emerged, we constructed Bayesian and maximum likelihood phylogenetic trees of DMRT family members without mammalian- and reptilian-specific *DMRT7* and mammalian-specific *DMRT8*. We analyzed the members in 19 species representing eight different phyla in bilaterians; Brachiopoda, Mollusca, Priapulida, Nematoda, Arthropoda, Hemichordata, Echinodermata and Chordata (Fig. 3; Fig. S2, and Table S1). Chordata included nine species from various taxa including Urochordata, Cephalochordata Chondrichthyes, Actinopterygii, Sarcopterygii, Amphibia and Mammalia. The DM domain regions, which are the only conserved regions among the family members in bilaterian animals, were used for the phylogenetic constructions. The DMRT1 cluster contained Dmrt1 orthologues and the paralogues encoded by the *O. latipes* and *X. laevis* sex-determining genes *dmy/dmrt1by* and *dmw*, respectively (Fig. 3; Fig. S2) (Matsuda et al., 2002; Nanda et al., 2002; Yoshimoto et al., 2008). As expected, there were no invertebrate genes in the DMRT1 cluster (Fig. 3). In addition, the phylogenetic trees indicate the following viewpoints. The DMRT2a/2b cluster included vertebrate Dmrt2 (Dmrt2a) and Dmrt2b, and invertebrate bilateria Dmrt2a/2b and arthropoda Dmrt11E. The DMRT3 cluster consisted of DMRT3 orthologues in deuterostomes. The DMRT4/5 cluster consisted of the vertebrata Dmrt4 and Dmrt5, invertebrate bilateria Dmrt4/5, arthropoda Dmrt99B and nematoda Dmd-5. The DMRT93B cluster consisted of Dmrt93B from most invertebrate bilateria, nematoda Dmd-4, and Dmrt93B from some fishes, suggesting that *dmrt93B* may have been lost during tetrapoda evolution. The DMRT6 cluster was comprised of only vertebrate DMRT6 orthologues. The Dsx and Mab-3 clusters consisted of arthropods and nematodes, respectively. These results indicated that *DMRT1* and *DMRT6* are vertebrate-specific genes. Accordingly, *dmrt1* and *dmrt6* genes might emerge through gene duplication during vertebrate evolution.

Interestingly, some *dmrt* genes including arthropod Dsx and nematode Mab-3 did not belong to the eight major subsets of DMRT in bilaterians. These diverged genes mediated through gene duplication might have evolved for taxa diversity. Especially, *C.* *elegans* and *C*. *remanei* possess many DMRT family members, which might have been derived from the high rate of spontaneous gene duplication in nematodes (Lipinski et al., 2011).

### *DMRT1* is a homolog but not an ortholog of arthropod *dsx* and nematode *mab-3*

**Fig. 4.**
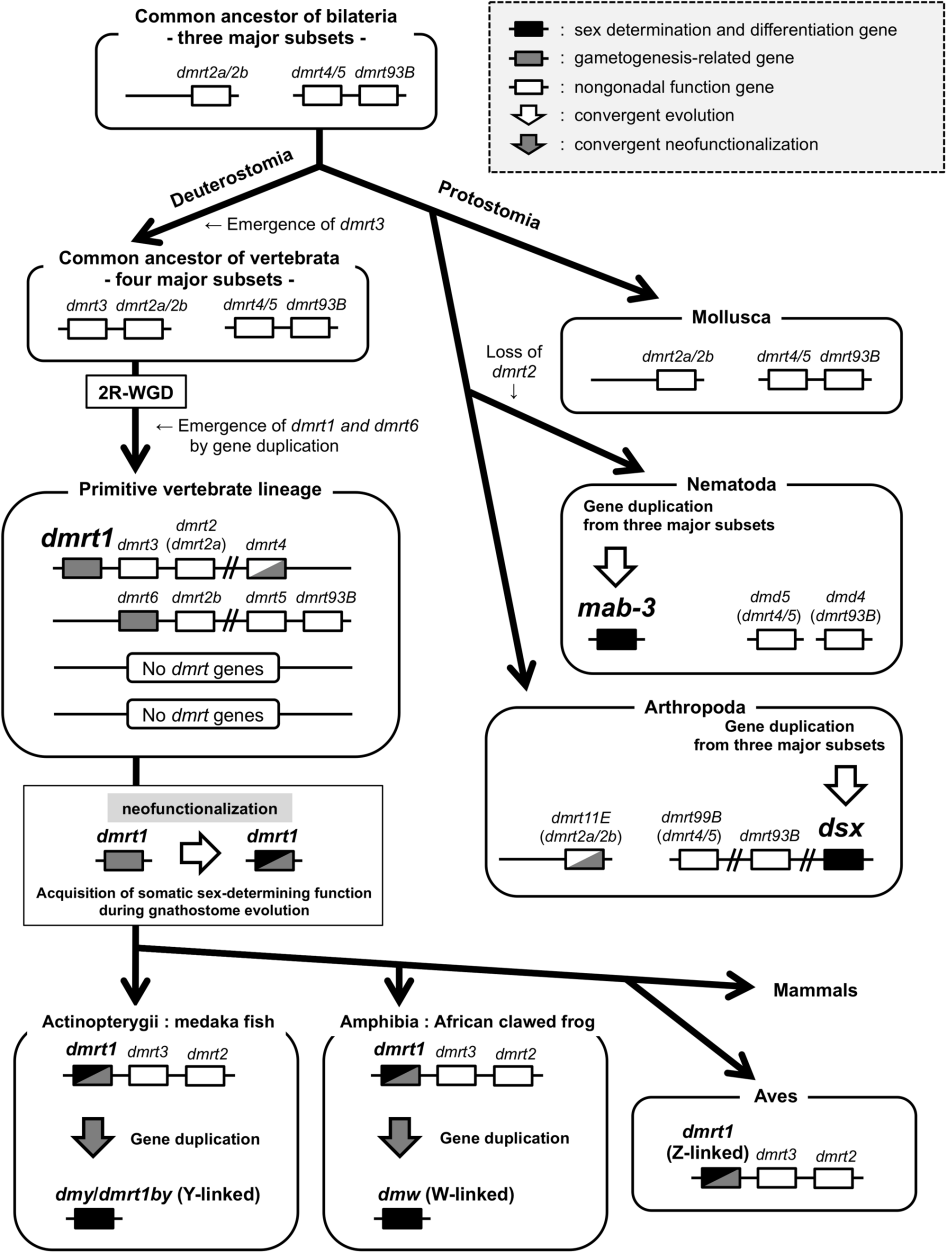
**Evolutionary history for the *DMRT* family genes in bilateria.** This figure was constructed based on figures in this study. The common ancestor of bilaterian animals possessed three ancestral genes, *dmrt2a/2b*, *dmrt4/5* and *dmrt93B*. In protostomia, sex-determining and/or primary sex-differentiating genes, arthropod *dsx* and nematode *mab-3* independently arose in each taxon. *dmrt3* could have emerged during deuterostome evolution. A common ancestor of vertebrata must have possessed four *DMRT* family genes, *dmrt2a/2b*, *dmrt3*, *dmrt4/5* and *dmrt93B*. The syntenies of *dmrt2a/2b*-*dmrt3* and *dmrt4/5*-*dmrt93B* are conserved before and after two rounds of whole genome duplication in the ancestral vertebrate. *dmrt1* gene might have emerged for germ-cell development in the primitive vertebrate lineage, and then acquired sex-determining function during gnathostome evolution. (Mawaribuchi et al., 2017a). Moreover, two sex-determining genes, the medaka fish *dmy*/*dmrt1by* and African clawed frog *dmw*, evolved independently through *dmrt1* duplication by convergent neofunctionalization. Other *DMRT* genes are not known to be involved in somatic sex determination and differentiation to date.

As mentioned in the Introduction section, there is no clear conclusion to the question whether *dmrt1*, *dsx* and *mab-3* are orthologous to one another or not. However, our recent report indicated that the ancestral vertebrate *dmrt1* gene might have emerged not for sex determination but for germ-cell development (Mawaribuchi et al., 2017a). Our syntenic and phylogenetic analyses in this study showed that a common ancestor of vertebrata must have possessed only four *DMRT* family genes, *dmrt2a/2b*, *dmrt3*, *dmrt4/5* and *dmrt93B* (Figs 1, 2, 3; Fig. S2). The syntenies of *dmrt2a/2b*-*dmrt3* and *dmrt4/5*-*dmrt93B* are conserved before and after 2R-WGD in a common ancestor of vertebrates (Figs 1 and 2). In addition, *dmrt1* and *dmrt6* might emerge in the primitive vertebrate lineage (Fig. 3; Fig. S2). Importantly, the *dsx* and *mab-3* genes have been found only in the subphylum Hexapoda among Arthropoda and the phylum Nematoda, respectively (Fig. 3; Fig. S2, and Table S1) (Price et al., 2015). These findings suggested that *DMRT1* is not orthologous to arthropod *dsx* and nematode *mab-3*. We then summarized molecular evolution of the *DMRT* gene family in bilateria (Fig. 4). *dsx*, *mab-3* and *Dmrt1* play important roles in sex determination and/or sex differentiation (Raymond et al., 2000; Smith et al., 2009; Matson and Zarkower, 2012). *Oryzias* *latipes* and *X. laevis* sex-determining genes, *dmy/dmrt1by* and *dmw*, independently evolved from duplication of *dmrt1* during the species diversity in each taxon (Matsuda et al., 2002; Nanda et al., 2002; Kondo et al., 2004; Bewick et al., 2011; Mawaribuchi et al., 2017b). Other *DMRT* genes have not been known to be involved in sex determination and sex differentiation to date. Then, we propose the independent evolution of *dmrt1* homologs, *dsx* and *mab-3* for sex determination and primary sex differentiation in the *DMRT* gene family.

## MATERIALS AND METHODS

### Sequence analysis

The *DMRT* gene sequences were obtained from the GenBank or various databases and genomes by BLAST (Table S1). Synteny analyses were also performed by BLAST using the obtained sequences and various genome sequences (Table S1). The protein sequences were aligned using MUSCLE (https://www.megasoftware.net), and gaps (insertions/deletions) were removed (Fig. S3). A best-fit protein substitution model was selected by Aminosan (https://www.fifthdimension.jp). Maximum likelihood and Bayesian phylogenetic analyses were performed using MEGA7 and MrBayes5D, respectively, with an rtREV+F+G model (https://www.megasoftware.net, https://www.fifthdimension.jp).

## Supplementary Material

Supplementary information
