# Supplementary material for: Independent evolution for sex determination and differentiation in the DMRT family in animals
Source: Biol Open. 2019 Aug 15;8(8):bio041962. doi: 10.1242/bio.041962 (PMC6737965; doi:10.1242/bio.041962)
Supplement: Supplementary information [file biolopen-8-041962-s1.pdf]

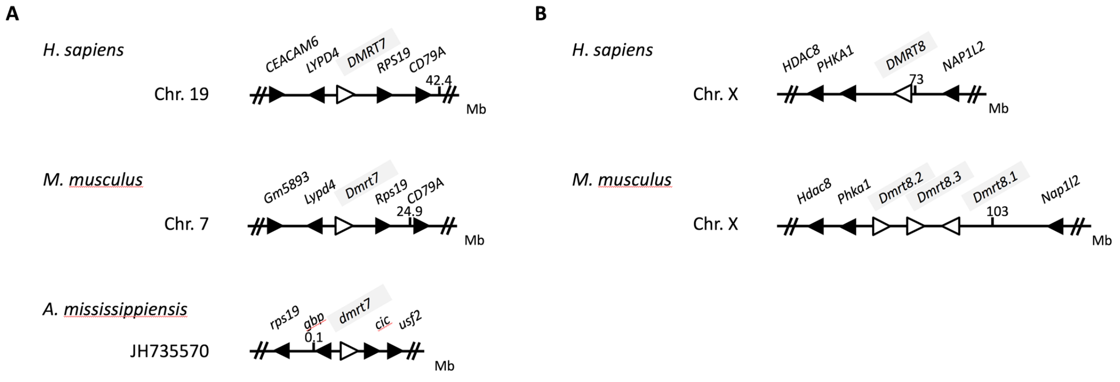

Figure S1. Synteny analysis of *DMRT7* and *DMRT8* genes in vertebrates.

(A) Synteny of *DMRT7*. (B) Synteny of *DMRT8*. Triangles indicate genes and their tips correspond to their 3′-ends. White and black triangles represent *DMRT* genes and the surrounding genes, respectively. Chr., Chromosome; LG, Linkage group; (-), reverse relationship.

Biology Open • Supplementary information

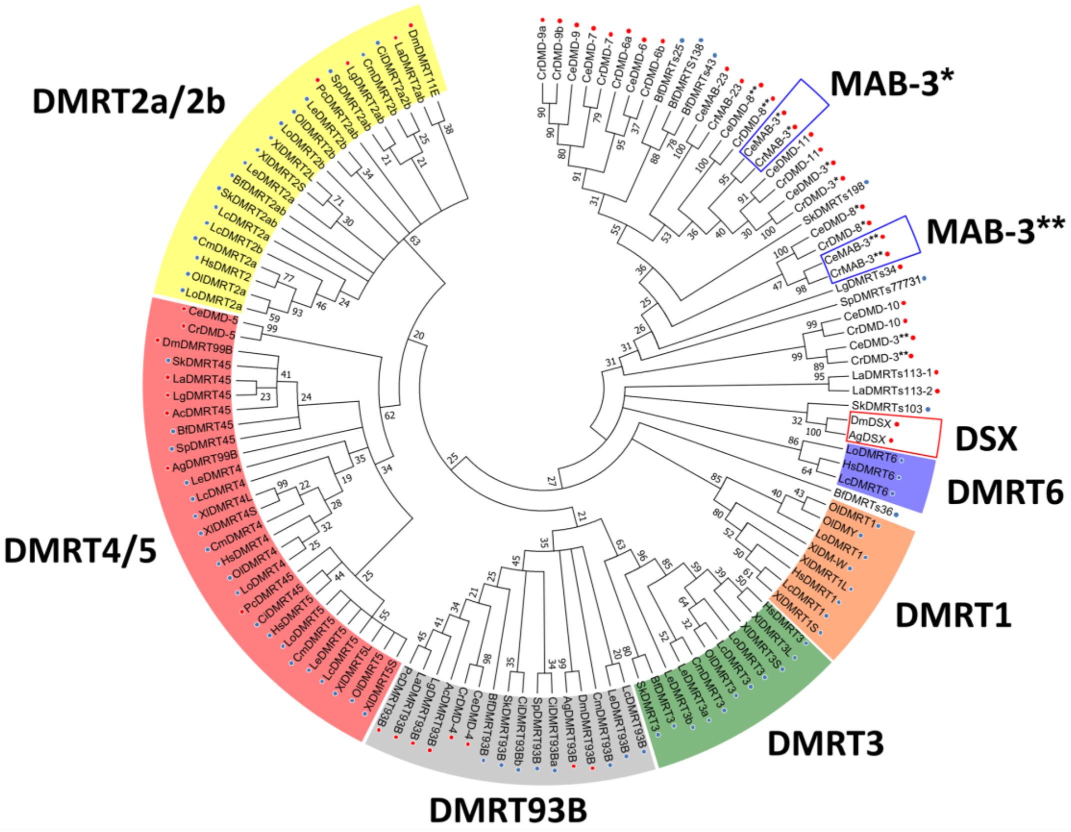

Figure S2. Maximum likelihood tree of bilaterian *DMRT* family genes. The tree was constructed by MEGA7 using the protein sequences of the DM domains from 19 species representing eight different phyla in bilateria (Fig. S3). Brachiopoda, *Lingula anatina* (La); Mollusca, *Aplysia californica* (Ac); Mollusca, *Lottia gigantea* (Lg); Priapulida, *Priapulus caudatus* (Pc); Nematoda, *Caenorhabditis elegans* (Ce); Nematoda, *Caenorhabditis remanei* (Cr); Arthropoda, *Anopheles gambiae* (Ag); Arthropoda, *Drosophila melanogaster* (Dm); Hemichordata, *Saccoglossus kowalevskii* (Sk); Echinodermata, *Strongylocentrotus purpuratus* (Sp); Chordata, Urochordata, *Ciona intestinalis* (Ci); Chordata, Cephalochordata, *Branchiostoma floridae* (Bf); Chordata, Vertebrata, Chondrichthyes, *Callorhinchus milii* (Cm); Chordata, Vertebrata, Chondrichthyes, *Leucoraja erinacea* (Le); Chordata, Vertebrata, Actinopterygii, *Lepisosteus oculatus* (Lo); Chordata, Vertebrata, Actinopterygii, *Oryzias*

Biology Open • Supplementary information

latipes (Ol); Chordata, Vertebrata, Sarcopterygii, *Latimeria chalumnae* (Lc); Chordata, Vertebrata, Amphibia, *Xenopus laevis* (Xl); Chordata, Vertebrata, Mammalia, *Homo sapiens* (Hs). Model test was performed by Aminosan (rtREV+F_Gamma). The bootstrap consensus tree was inferred from 100 replicates. Blue and red circles represent Deuterostomia and Protostomia, respectively. * and ** indicate DM domain regions on 5' and 3' sides, respectively. The numbers indicate bootstrap values. The values less than 20 % were collapsed.

Biology Open • Supplementary information

A.californica_DMRT4/5

RTPKCARCRNHGVVSALKGHYCRWRDCVCAKCTLIAERQRVMAAQVALRRQQAQEENEARELG

A.californica_DMRT93B

RKPKCARCRNHGMVSWLKGHHCQYKDCTCPKCNLIAERQRVMAAQVALKRQQAAEDAIALGIR

A.gambiae_DMRT93B

RVPKCARCRNHGVISGLRGHLCSYRNCRCAKCELILSRQKIMAAQVALKRQQAVEDAIALRLA

A.gambiae_DMRT99B

RTPKCARCRNHGVVSALKGHYCRWRDCVCAKCTLIAERQRVMAAQVALRRQQAQEENEVRDLG

A.gambiae_DSX

TPPNCARCRNHGLKIGLKGHYCKYRACQCEKCCLTAERQRVMALQTALRRAQTQDEQRALNEG

B.floridae_DMRT_Scaffold_138

REYTCKRCRVHGVIVAVKGHQCPWKHCQCPGCQQVTLYRQEHASEINARRGGSDISAADVINI

B.floridae_DMRT_Scaffold_25

REYHCKKCRVHGIEVPLKGHKCPWDKCGCDGCLQVVSYRRQHVHDGRAMTASDVINIANLSRK

B.floridae_DMRT_Scaffold_36

RPPMCARCRNHGVSALLKGHKCQWRDCECPKCYLIVERRRVMAAQVALRRAQDAEDKSRQSTP

B.floridae_DMRT_Scaffold_43

REYTCKRCRVHGVVVAVKGHQCHWKYCQCPGCQQVTSYRQEHASEINAKREVGSDMSVADVIN

B.floridae_DMRT2a/b

RTPKCARCRNHGVVSCLKGHYCRWRDCQCANCLLVVERQRVMAAQVALRRQQATDLGRGGGTA

B.floridae_DMRT3

RTPKCARCRNHGVLSWLKGHYCRFKDCMCEKCILIAERQRVMAAQVALRRQQAQEQSILQQFG

B.floridae_DMRT4/5

RTPKCARCRNHGVVSALKGHYCRWRDCMCAKCTLIAERQRVMAAQVALRRQQAQEENEARELG

B.floridae_DMRT93B

RRPKCARCRNHGIVSWLKGHHCRYRDCTCPKCNLIAERQRVMAAQVALKRQQAAEDAIAMGLR

C.elegans_DMD-10

RVPNCQKCGQHGRKSRLKGHSCPFRECPCAKCAVVSERQKLMADQIKIRRRQRKDTLLTFAKN

C.elegans_DMD-11

KIYYCQRCLNHDVPRPRKNHECPYADCTCEKCGLVEKRRILNIRLQNYNQFNIENENDSKLII

C.elegans_DMD-3*

RVYYCQRCLNHGLREKRKNHSCTFRFCQCSNCIMVERRRQLNSRLMQIDGSRDEKPMTTLTMA

C.elegans_DMD-3**

Biology Open • Supplementary information

RRPNCQRCAQHSVVNRLKGHACPFRDCFCAKCQVVVERQKLMADQIKLRRRQKREKNNLNSER

C.elegans_DMD-4

RKPKCARCRNHGLVSWLKGHHCKYKECACEKCNLIAERQRVMAAQVALKRRQATEDAIALGLR

C.elegans_DMD-5

RTPKCARCRNHGTTSALKGHYCQWKDCMCAKCTLIAERQRVMAAQVALRRQQSQEERDARDLE

C.elegans_DMD-6

RILYCRKCEGHGEKVILKNHQCPYILCNCKSCEKLNYKRLKSFNKRNKEKIELAAALNAKRHA

C.elegans_DMD-7

RTLFCRKCEGHGQQVVLKGHRCPFNNCSCKTCTNVMSMRANAIIRRYRTRTLEGGLVLKPVHF

C.elegans_DMD-8*

VKRHCGMCKQHGVFVETRGHTCEYRSCECEQCDLVRKRREIMSTQIRLRREQDKKFQRTNDIS

C.elegans_DMD-8**

MCYFCQKCKNHNVLVWKKNHECQYSSCECQQCNLIDSRRALDRHIKKRKMSIKGNTVEAIAPK

C.elegans_DMD-9

KKLTCRKCEGHGTYAILKGHVCPYKDCSCGTCASVMSMRANALIRRFRHRQPDQSMAVVKALR

C.elegans_MAB-23

EQYMCQLCANHGIFNPKKGHKCPYRTCPCSLCALNTKRRALDQIERQLKHTNEPMTGGQTATSM

C.elegans_MAB-3*

KNYYCQRCLNHGELKPRKGHDCRYLKCPCRECTMVEQRRQLNNLLSKKKIHCTPATQTRDGKR

C.elegans_MAB-3**

RDPHCARCSAHGVLVPLRGHMCQFVTCECTLCTLVEHRRNLMAAQIKLRRSQQKSRDGKEPKR

C.intestinalis_DMRT2a/2b

RTPKCARCRNHGVVSTLKGHHCRWRDCQCSNCLLVVERQRIMAAQQAADVKKSGRSDSETSSP

C.intestinalis_DMRT4/5

RTPKCARCRNHGVVSALKGHYCRWKDCLCPKCTLIAERQRVMAAQVALRRQQAQEENETRELR

C.intestinalis_DMRT93Ba

RKPKCARCRNHGMISWLKGHHCPYRDCACAKCNLIAERQKVMAAQVALKRQQAAEDAIALGLR

C.intestinalis_DMRT93Bb

RKPKCARCRNHGVISWLKGHRCPHRECTCAKCNLIAERQRVMAAQVALKRQQAAEDAIALGLK

C.milii_DMRT2a

RTPKCARCRNHGVVSCLKGHFCRWRDCQCANCLLVVERQRVMAAQVALRRQQATEDKKGLSGK

C.milii_DMRT2b

RTPKCARCRNHGVVSGLKGHCCRWRECECSNCLLVLERQRIMAAQVALRRQQCSQEKRDSAAL

Biology Open • Supplementary information

C.milii_DMRT3

RTPKCARCRNHGVLSWLKGHYCRFKDCTCDKCILIIERQRVMAAQVALRRQQANECIGSLIPE

C.milii_DMRT4

RTPKCARCRNHGVVSALKGHFCRWRDCLCAKCMLIAERQRVMAAQVALRRQQAQEESEVRELQ

C.milii_DMRT5

RTPKCARCRNHGVVSALKGHYCRWKDCMCAKCTLIAERQRVMAAQVALRRQQAQEENEARELQ

C.milii_DMRT93B

RKPKCARCRNHGVLAWLKGHLCPYSGCACVKCILISERRRVMAAQVALRRQQAVEDAMSLGCF

C.remanei_DMD-10

RVPNCQKCGQHGRKSRLKGHNCPFRECPCAKCAVVTERQKLMADQIKIRRRQRKDTLMNFTRE

C.remanei_DMD-11

KIYYCQRCLNHDTPRPRKNHECPYADCTCDKCGLVEKRRILNIRLQNYNTVENDFDGPSPIDD

C.remanei_DMD-3*

RVYYCQRCLNHGLREKRKNHSCSFRFCQCSNCIMVERRRQLNSRLMQIEGSQEDKKPSPPTPL

C.remanei_DMD-3**

RRPNCQRCAQHSVVNRLKGHACPFRDCFCAKCQVVVERQKLMADQIKLRRRQKREKNNLNSER

C.remanei_DMD-4

RKPKCARCRNHGLVSWLKGHHCKYKECACEKCNLIAERQRVMAAQVALKRRQATEDAIALGLR

C.remanei_DMD-5

RTPKCARCRNHGTVSALKGHYCRWKDCMCAKCTLIAERQRVMAAQVALRRQQSQEEKDARDLE

C.remanei_DMD-6a

RILYCRKCEGHGEKVILKNHQCPYILCNCKSCEKLNYKRLKSFNKRNKEKIELAAALNAKRHA

C.remanei_DMD-6b

RILYCRKCEGHGEKVILKNHQCPYILCNCKSCEKLNYKRLKSFNKRNKEKIELAAALNAKRHA

C.remanei_DMD-7

RTLFCRKCEGHGQQVVLKGHRCPFNNCSCKTCTNVMSMRANAIIRRYRTRTLEGGLVLKPVHF

C.remanei_DMD-8*

VKRHCGMCKQHGVIVETRGHTCEYKNCSCEHCELVRKRREIMSTQIRLRREQDKKFQRTTDIN

C.remanei_DMD-8**

MCYFCQKCKNHNVLVWKKNHECQYKDCDCEQCNLIDSRRALDRHIKKRKMNMKENGVGATAPK

C.remanei_DMD-9a

KRLTCRKCEGHGLYAILKGHVCPYKDCSCGTCASVMSMRANALIRRFRHRQPDKSMAVVKSLR

C.remanei_DMD-9b

Biology Open • Supplementary information

KRLTCRKCEGHGLYAILKGHVCPYKDCSCGTCASVMSMRANALIRRFRHRQPDKSMAVVKALR

C.remanei_MAB-23

EQYMCQLCANHGIFNPKKGHKCPYRTCPCSLCALNTKRRALDQIERQLKHTNEPMVAHTPTSM

C.remanei_MAB-3*

KNYYCQRCLNHGEYKPRKGHDCRYLQCPCAECTMVERRRQLNNMLSKKKVHCAPNTQTRDGKR

C.remanei_MAB-3**

RDPHCARCSAHGVLVPLRGHMCQFVTCTCTLCALVENRRMLMAAQIKLRRSQQKTRDGKEPKI

D.melanogaster_DMRT11E

RTPKCARCRNHGVISCVKGHLCRWRECCCPNCQLVVDRQRVMAAQVALRRQQTMEALEATASS

D.melanogaster_DMRT93B

RVPKCARCRNHGIISELRGHLCTYKNCKCAKCVLIFERQRIMAAQVALKRQQAVEDAIAMRLV

D.melanogaster_DMRT99B

RTPKCARCRNHGVVSALKGHYCRWRDCVCAKCTLIAERQRVMAAQVALRRQQAQEENEARELG

D.melanogaster_DSX

TPPNCARCRNHGLKITLKGHYCKFRYCTCEKCRLTADRQRVMALQTALRRAQAQDEQRALHMH

H.sapiens_DMRT1

RLPKCARCRNHGYASPLKGHFCMWRDCQCKKCNLIAERQRVMAAQVALRRQQAQEEELGISHP

H.sapiens_DMRT2

RTPKCARCRNHGVVSCLKGHFCRWRDCQCANCLLVVERQRVMAAQVALRRQQATEDKKGLSGK

H.sapiens_DMRT3

RTPKCARCRNHGVLSWLKGHYCRFKDCTCEKCILIIERQRVMAAQVALRRQQANESLESLIPD

H.sapiens_DMRT4

RTPKCARCRNHGVVSALKGHFCRWRDCACAKCTLIAERQRVMAAQVALRRQQAQEESEARGLQ

H.sapiens_DMRT5

RTPKCARCRNHGVVSALKGHYCRWKDCLCAKCTLIAERQRVMAAQVALRRQQAQEENEARELQ

H.sapiens_DMRT6

RTPKCSRCRNHGFLVPVKGHKCRWKQCLCEKCYLISERQKIMAAQKVLKTQAAEEEQEAALCA

L.anatina_DMRT_scaffold113-1

RSPKCARCRNHGVCQPLKGHYCQFRECSCNSCLLIKERQKIMAKQVALKRHQELDETMGLQEY

L.anatina_DMRT_scaffold113-2

RSPKCARCRNHGVCQPLKGHYCQFRECSCNSCLLIKERQKIMAKQVALKRHQELDETMGLQEY

L.anatina_DMRT2ab

RTPKCARCRNHGVISCLKGHYCRWRDCSCPNCLLVVERQRVMAAQVALRRHQTTEINKTLKAK

Biology Open • Supplementary information

L.anatina_DMRT4/5

RTPKCARCRNHGVVSALKGHYCRWRDCVCAKCTLIAERQRVMAAQVALRRQQAQEENEARELG

L.anatina_DMRT93B

RRPKCARCRNHGMVSWLKGHHCRFKDCSCAKCNLIAERQRVMAAQVALKRQQAAEDAIAMGLR

L.chalumnae_DMRT1

RLPKCARCRNHGYASPLKGHFCMWRDCQCKKCSLIAERQRVMAAQVALRRQQAQEEELGISHP

L.chalumnae_DMRT2a

RTPKCARCRNHGVVSCLKGHFCRWRDCQCANCLLVVERQRVMAAQVALRRQQATEKKKNSEMK

L.chalumnae_DMRT2b

RSPKCARCRNHGVVSCLKGHFCRWKDCQCANCLLVVERQRVMAAQVALRRQQATEIKKGVSTK

L.chalumnae_DMRT3

RTPKCARCRNHGVLSWLKGHYCRFKDCTCEKCILIIERQRVMAAQVALRRQQANESLESLIPE

L.chalumnae_DMRT4

RTPKCARCRNHGVVSALKGHFCRWRDCVCAKCTLIAERQRVMAAQVALRRQQAQEENEVRELQ

L.chalumnae_DMRT5

RTPKCARCRNHGVVSALKGHYCRWKDCMCAKCTLIAERQRVMAAQVALRRQQAQEENEARELQ

L.chalumnae_DMRT6

RTPKCSRCRNHGFIIPLKGHKCNWKQCQCEKCSLITERQKIMAAQKVLKKQQQKEDELAGNEG

L.chalumnae_DMRT93B

RKPKCARCRNHGLIAWLKGHLCPYRDCGCPKCKLIAERQRVMAAQVALRRQQAAEDVIALGLR

L.erinacea_DMRT2a

RTPKCARCRNHGVVSCLKGHFCRWRDCQCANCLLVVERQRVMAAQVALRRQQATEVSWDPRGA

L.erinacea_DMRT2b

RSPKCARCRNHGVVSCLKGHFCRWRDCQCTNCLLVVERQRVMAAQVALRRQQAIEVRLYGKKT

L.erinacea_DMRT3a

RTPKCARCRNHGVLSWLKGHYCRFKDCTCEKCILIIERQRVMAAQVALRRQQANECIGSLIPD

L.erinacea_DMRT3b

RTPKCARCRNHGVLSWLKGHYCRFKDCTCDKCILILERQRVMAAQVALRRQQSNENPCSLVLP

L.erinacea_DMRT4

RTPKCARCRNHGVVSALKGHFCRWRDCMCAKCMLIAERQRVMAAQVALRRQQAQEENEARELQ

L.erinacea_DMRT5

RTPKCARCRNHGVVSALKGHYCRWKDCMCAKCTLIAERQRVMAAQVALRRQQAQEENEARELQ

L.erinacea_DMRT93B

Biology Open • Supplementary information

RVPKCARCRNHGVIAWLKGHLCPFRDCSCAKCILISERQRVMAAQVALKRRQAAEEVVALGLR

L.gigantea_DMRT_Scaffold_34

KRPCCSKCRNHCVLSPLKGHRCLYKDCQCPKCQLVEARRDISKRQIALRRLQIEEENYGSSPA

L.gigantea_DMRT2a/b

RTPKCARCRNHGVVSCLKGHFCRWRDCQCSNCLLVVERQRIMAAQVSLRRHQASDMTGALKAK

L.gigantea_DMRT4/5

RTPKCARCRNHGVVSALKGHYCRWRDCVCAKCTLIAERQRVMAAQVALRRQQAQEENEARELG

L.gigantea_DMRT93B

RKPKCARCRNHGMVSWLKGHHCGFKDCDCAKCNLIAERQRVMAAQVALKRQQAAEDAIAMGIR

L.oculatus_DMRT1

RKPKCSRCRNHGYESPLKGHFCNWRDCQCEKCKLIAERQRVMAAQVALRRQQAQEEEMGICSL

L.oculatus_DMRT2a

RTPKCARCRNHGVVSCLKGHFCRWRDCQCANCLLVVERQRVMAAQVALRRQQATEDKKGIAGK

L.oculatus_DMRT2b

RSPKCARCRNHGVVSCLKGHFCRWRDCRCTNCLLVVERQRVMAAQVALRRQQATEVKKDANGI

L.oculatus_DMRT3

RTPKCARCRNHGVLSWLKGHYCRFKDCTCEKCILIIERQRVMAAQVALRRQQANESLESLIPE

L.oculatus_DMRT4

RTPKCARCRNHGVVSALKGHFCRWRDCVCAKCTLIAERQRVMAAQVALRRQQAQEESEARELQ

L.oculatus_DMRT5

RTPKCARCRNHGVVSALKGHYCRWKDCMCAKCTLIAERQRVMAAQVALRRQQAQEENEARELQ

L.oculatus_DMRT6

RTPKCARCRNHGFIVQLKGHKCPFYHCSCWKCSLITERTKIMASQRRLKKLQNEEALGNANLT

O.latipes_DMRT1

RMPKCSRCRNHGFVSPLKGHFCRWKDCRCAKCKLIAEGQRVMAAQVALRRQQAQEEELGICSP

O.latipes_DMRT2a

RTPKCARCRNHGVVSCLKGHFCRWRDCQCANCLLVVERQRVMAAQVALRRQQATEDKKGISGK

O.latipes_DMRT2b

RSPKCARCRNHGVVSCLKGHFCRWRDCRCACCLLVVERQRVMAAQVALRRQQAAEVRRVPGQS

O.latipes_DMRT3

RTPKCARCRNHGVLSWLKGHYCRFKDCTCEKCILIIERQRVMAAQVALRRQQANESLESLIPE

O.latipes_DMRT4

RTPKCARCRNHGVVSALKGHFCRWRDCVCAKCTLIAERQRVMAAQVALRRQQAQEESEARDLR

Biology Open • Supplementary information

O. latipes_DMRT5

RTPKCARCRNHGVVSALKGHYCRWKDCMCAKCTLIAERQRVMAAQVALRRQQAQEENEARELQ

O. latipes_DMY

RVPKCSRCRNHGLKTPLKGHFCRWKDCQCFKCEQIMVRQRVMAAQVADRRQQAQEEELGICSP

P. caudatus_DMRT2ab

RTPKCARCRNHGVVSCLKGHYCRWRDCQCANCLLVVERQRIMAAQVALRRQQSQEQKKDGETE

P. caudatus_DMRT4/5

RTPKCARCRNHGVVSALKGHYCRWKDCVCAKCTLIAERQRVMAAQVALRRQQAQEENEARELG

P. caudatus_DMRT93B

RKPKCARCRNHGMVSWLKGHHCRFKDCTCAKCNLIAERQRVMAAQVALKRQQAAEDAIAMGLR

S. kowalevskii_DMRT_Scaffold_103

PKPKCARCRNHGIVIRIKDHVCCYRNCTCQDCNLTKMRQDVMKKQVALRRRQATDRRIGISRT

S. kowalevskii_DMRT2a/b

RTPKCARCRNHGVVSCLKGHYCRWRDCQCANCLLVVERQRVMAAQVALRRQQATEVSKGTKTK

S. kowalevskii_DMRT3

RQPKCARCRNHGVLSWLKGHYCRFKECMCEKCILIAERQRVMAAQVALRRQQAHEQSILYQYQ

S. kowalevskii_DMRT4/5

RTPKCARCRNHGVVSALKGHYCRWRDCVCAKCTLIAERQRVMAAQVALRRQQAQEENEARELG

S. kowalevskii_DMRT93B

RKPKCARCRNHGVISWLKGHHCRFRECTCPKCNLIAERQRVMAAQVALKRQQAAEDAIALGLR

S. kowalevskii_Scaffold_198

RMPTCTRCRYHDIDIPLKGHHCPYKECECSRCGLIMKRRMINVAQQELHRQHNPKKSVLNAEN

S. purpuratus_DMRT_Scaffold_1353

RHPTCARCRNHGLILDLKGHLCEYRYCRCTRCIVVSQRRVVMAKQVALSREQVRQHRQDQDTS

S. purpuratus_DMRT2a/b

RTPKCARCRNHGVVSCLKGHFCRWRDCRCTNCLLVVERQRVMAAQVALRRQQSSDPGSGNAAG

S. purpuratus_DMRT4/5

RTPKCARCRNHGVVSALKGHYCRWRDCICAKCTLIAERQRVMAAQVALRRQQAQEENEAKELG

S. purpuratus_DMRT93B

RKPKCARCRNHGMISWLKGHHCRFRDCRCAKCNLIAERQRVMAAQVALKRQQAAEDAIILGLR

X. laevis_DMRT1.L

RLPKCARCRNHGYASPLKGHYCMWRDCQCKKCSLIAERQRVMAAQVALRRQQAQEEELGISHP

X. laevis_DMRT1.S

Biology Open • Supplementary information

RLPKCARCRNHGYASPLKGHFCMWRDCQCKKCSLIAERQRVMAAQVALRRQQAQEEELGISHP

X.laevis_DMRT2.L

RTPKCARCRNHGVVSCLKGHFCRWRDCQCANCLLVVERQRVMAAQVALRRQQATEVSDKGDHS

X.laevis_DMRT2.S

RTPKCARCRNHGVVSCLKGHFCRWRDCQCANCLLVVERQRVMAAQVALRRQQATEVSSKADMY

X.laevis_DMRT3.L

RTPKCARCRNHGVLSWLKGHYCRFKDCTCEKCILIIERQRVMAAQVALRRQQANESLESLIPD

X.laevis_DMRT3.S

RTPKCARCRNHGVLSWLKGHYCRFKDCSCEKCILIIERQRVMAAQVALRRQQANESLESLIPD

X.laevis_DMRT4.L

RTPKCARCRNHGVVSALKGHFCRWRDCSCAKCTLIAERQRVMAAQVALRRQQAQEECEVRDVQ

X.laevis_DMRT4.S

RTPKCARCRNHGVVSALKGHFCRWRDCSCAKCTLIAERQRVMAAQVALRRQQAQEECEVRDVQ

X.laevis_DMRT5.L

RTPKCARCRNHGVVSALKGHYCRWKDCMCAKCTLIAERQRVMAAQVALRRQQAQEENEARELQ

X.laevis_DMRT5.S

RTPKCARCRNHGVVSALKGHYCRWKDCMCAKCTLIAERQRVMAAQVALRRQQAQEENEARELQ

X.laevis_DM-W

RLHKCARCRNHGYATPLKGHFCIWRDCQCQKCSLITERQRVIAAQVALQRQQAQEEELGIYHP

Figure S3. Alignment of DM domains in bilateria used in Figure 3 and Figure S2.

Biology Open • Supplementary information

Table S1. List of *DMRT* gene family used in this study

Species	Gene name	Subset	DM domain region (Chr. or scaffold, start-end, strand)	Accession number, gene ID, protein ID
Lingula anatina (Brachiopoda)			LinAna1.0	
	dmrt2a/2b	2a/2b	scaffold185, 154685-158196, +	XM_013541850
	dmrt4/5	4/5	scaffold577, 160785- 160606, -	XM_013564040
	dmrt93B	93B	scaffold13, 111509- 111330, -	XM_013534373
	dmrt scaffold133-1	-	scaffold113, 385280- 385459, +	XM_013559056
	dmrt scaffold133-2	-	scaffold113, 514603- 514424, -	XM_013559084
Aplysia californica (Mollusca)			Broad2.0/Aplcal1.0	
	dmrt2a/2b	2a/2b	13, †-†, +	XP_012939103
	dmrt4/5	4/5	57, 475836-476039, +	XP_005096931
	dmrt93B	93B	57, 651905-653217, +	XP_005096932
Lottia gigantean (Mollusca)			JGI v1.0	
	dmrt2a/2b	2a/2b	53, 1412569-1406365, -	jgi|Lotgi1|80701|gw1.53.174.1
	dmrt4/5	4/5	15, 1242558-1242355, -	jgi|Lotgi1|80706|gw1.15.347.1
	dmrt93B	93B	15, 1269871-1270074, +	jgi|Lotgi1|60671|gw1.15.80.1
	dmrt scaffold34	-	34, 235849-236052, +	ESO92515
Priapulus caudatus (Priapulida)			-	
	dmrt2a/2b	2a/2b	-	XM_014820073
	dmrt4/5	4/5	-	XM_014810247
	dmrt93B	93B	-	XM_014819542

Species	Gene name	Subset	DM domain region (Chr. or scaffold, start-end, strand)	Accession number, gene ID, protein ID
			WS220/ce10	
Caenorhabditis elegans (Nematoda)	*dmd-3**	-	V, 19654435-19654658, -	NP_001256882
	*dmd-3***	-	V, 19651735-19651932, -	NP_001256882
	dmd-4	93B	X, 14858748-14858718, -	NP_510466
	dmd-5	4/5	II, 5773508-5774073, -	NP_495138
	dmd-6	-	IV, 14619538-14619795, +	NP_502770
	dmd-7	-	V, 6250302-6250505, +	NP_741551
	*dmd-8**	-	V, 335975-336148, -	NP_503176
	*dmd-8***	-	V, 335644-335847, -	NP_503176
	dmd-9	-	IV, 3254119-3254390, -	NP_500305
	dmd-10	-	V, 13223366-13225775, -	NP_506288
	dmd-11	-	V, 13236193-13236442, -	NP_506289
	*mab-3**	-	II, 9733423-9735751, +	NP_001022464
	*mab-3***	-	II, 9735746-9736321, +	NP_001022464
	mab-23	-	V, 10633037-10634071, +	NP_001041089
			-	
Caenorhabditis remanei (Nematoda)	*dmd-3**	-	-	XM_003095962
	*dmd-3***	-	-	XM_003095962
	dmd-4	93B	-	XM_003106193
	dmd-5	4/5	-	XM_003108836
	dmd-6a	-	-	XM_003096940
	dmd-6b	-	-	XM_003089040
	dmd-7	-	-	XM_003112356
	*dmd-8**	-	-	XM_003116105
	*dmd-8***	-	-	XM_003116105
	dmd-9a	-	-	XM_003102651
	dmd-9b	-	-	XM_003102760
	dmd-10	-	-	XM_003112367
	dmd-11	-		XM_003112462
	*mab-3**	-	-	XM_003094435
	*mab-3***	-	-	XM_003094435
	mab-23	-	-	XM_003101397

Biology Open • Supplementary information

Species	Gene name	Subset	DM domain region (Chr. or scaffold, start-end, strand)	Accession number, gene ID, protein ID
Anopheles gambiae (Arthropoda)			-	
	dmrt93B	93B	-	XM_321748
	dmrt99B	4/5	-	XM_310668
	dsx	-	-	XM_003436649
Drosophila melanogaster (Arthropoda)			BDGP Release5/dm3	
	dmrt11E	2a/2b	X, 13262817-13263020, +	NP_511146
	dmrt93B	93B	3R, 16899834-16901586, +	NP_524549
	dmrt99B	4/5	3R, 25518628-25519201, +	NP_524428
	dsx	-	3R, 3786216-3786419, -	NP_731197
Strongylocentrotus purpuratus (Echinodermata)			Baylor 2.1/strPur2	
	dmrt2a/2b	2a/2b	84487, 46479-46673, +	SPU_008648
	dmrt4/5	4/5	10416, 349878-350072, -	XP_786938
	dmrt93B	93B	22986, 66944-67138, +	XP_003723549
	dmrt scaffold77731	-	77731, 47536-47342, -	XP_795079
Saccoglossus kowalevskii (Hemichordata)			JGI v3.0	
	dmrt2a/2b	2a/2b	10, 1853565-1853362, -	XP_002732510
	dmrt3	3	10, 1834203-1834406, +	XP_006813729
	dmrt4/5	4/5	203, 229755-229958, +	Sakowv30011616m
	dmrt93B	93B	203, 272704-272907, +	NP_001164688
	dmrt scaffold103	-	103, 550342-550139, -	XP_006821777
	dmrt scaffold198	-	198, 172784-173133, +	Sakowv30006435m
Ciona intestinalis (Chordata, Urochordata)			JGI2.1/ci2	
	dmrt2a/2b	2a/2b	08q, 338052-335971, -	XP_002124643
	dmrt4/5	4/5	05q, 5020356-5018507, -	NP_001071680
	dmrt93Ba	93B	10q , 800624-801020, +	XP_004226593
	dmrt93Bb	93B	Scaffold141, 271842-271535, -	jgi|Cioin2|257512|gw1.141.27.1

Biology Open • Supplementary information

Species	Gene name	Subset	DM domain region (Chr. or scaffold, start-end, strand)	Accession number, gene ID, protein ID
			JGI v2.0	
Branchiostoma floridae (Chordata, Cephalochordata)	*dmrt2a/2b*	2a/2b	69, 604435-604638, +	XP_002605980
	dmrt3	3	69, 630916-630713, -	XP_002605981
	dmrt4/5	4/5	22, 364713-364916, +	XP_002611012
	dmrt93B	93B	22, 343978-344181, +	XP_002611011
	dmrt scaffold25	-	25, 508557-508354, -	XP_002610531
	dmrt scaffold36	-	36, 2099777-2100082, +	XP_002608975
	dmrt scaffold43	-	43, 2453868-2453665, -	XP_002607475
	dmrt scaffold138	-	138, 2389248-2389451, +	XP_002600009
			Callorhinchus_milii-6.1.3/calMil1	
Callorhinchus milii (Chordata, Vertebrata, Chondrichthyes)	*dmrt1*	1	KI635878, †-†, -	ACI43913
	dmrt2a	2a/2b	KI635878, 7137630-7139436, -	ACI43914
	dmrt2b	2a/2b	KI636088, 327034-327915, +	ACI43917
	dmrt3	3	KI635878, 7174209-7174412, -	XP_007890813
	dmrt4	4/5	KI635929, 2688584-2688787, -	ACI43916
	dmrt5	4/5	KI636278, 62199-62402, -	XP_007909461
	dmrt93B	93B	KI635929, 2672389-2672592, -	XP_007898235
			SkateBase Contigs Build 2	
Leucoraja erinacea (Chordata, Vertebrata, Chondrichthyes)	*dmrt1*	1	Contig642762	-
	dmrt2a	2a/2b	Contig94359	-
	dmrt2b	2a/2b	Contig713701	-
	dmrt3a	3	Contig71737, Contig2669513	-
	dmrt3b	3	Contig33532, Contig1640763	-
	dmrt4	4/5	Contig518, Contig49555	-
	dmrt5	4/5	Contig36209	-
	dmrt6p	6	Contig2696256	-
	dmrt93	93B	Contig1746182, Contig2689687	-

Species	Gene name	Subset	DM domain region (Chr. or scaffold, start-end, strand)	Accession number, gene ID, protein ID
			Ensembl LepOcu1	
Lepisosteus oculatus (Chordata, Vertebrata, Actinopterygii)	*dmrt1*	1	LG2, 47294138-47296792, +	XP_006627185
	dmrt2a	2a/2b	LG2, 47361545-47362404, +	XP_006627187
	dmrt2b	2a/2b	LG10, 9451949-9451029, -	XP_006634975
	dmrt3	3	LG2, 47341767-47341970, +	XP_006627186
	dmrt4	4/5	LG4, 58659622-58659825, +	XP_006630066
	dmrt5	4/5	LG10, 22142277-22142074, -	XP_006635098
	dmrt6	6	LG10, 9461487-9461284, -	ENSLOCP00000007844
			ASM223467v1 [NIG/UT MEDAKA1/oryLat2]	
Oryzias latipes (Chordata, Vertebrata, Actinopterygii)	*dmrt1*	1	9, 1213013-1213129, - [9 (ultracontig107), 170380-171541, +]	AAL02165
	dmrt2a	2a/2b	9, 1157368-1157538, - [9 (ultracontig107), †-†, +]	AAL02163
	dmrt2b	2a/2b	4, 16779620-16779811, + [4, 17897689-17898576, +]	XP_004068077
	dmrt3	3	9, 1175820-1176014, - [9 (ultracontig107), †-†, +]	AAL02164, AP006154
	dmrt4	4/5	18, 8179356-8179550, + [18, 7456565-7456768, +]	BAB63259
	dmrt5	4/5	4, 31232501-31232617, - [†, †-†, †]	Q76L87
	dmy	1	†, †-†, † [Scaffold1535, 24501-25411, +]	AAN05398

Biology Open • Supplementary information

Species	Gene name	Subset	DM domain region (Chr. or scaffold, start-end, strand)	Accession number, gene ID, protein ID
			Ensembl v1.71	
Latimeria chalumnae (Chordata, Vertebrata, Sarcopterygii)	*dmrt1*	1	JH127237, 847919-834083, -	XP_006001259
	dmrt2a	2a/2b	JH127237, 616422-614091, -	XP_014347041
	dmrt2b	2a/2b	JH130928, 47168-45344, -	XP_006013578
	dmrt3	3	JH127237, 678252-678049, -	XP_006001258
	dmrt4	4/5	JH127118, 444316-444519, +	XP_005999966
	dmrt5	4/5	JH127555, 670295-670092, -	XP_006004087
	dmrt6	6	JH130928, 92442-92239, -	XP_006013577
	dmrt93B	93B	JH127118, 463305-463508, +	-
			Xenbase v7.1	
Xenopus laevis (Chordata, Vertebrata, Amphibia)	*dmrt1.L*	1	1L (scaffold106782), 2260465-2257264, -	NP_001089969
	dmrt1.S	1	1S (scaffold214452), 257488-267250, +	NP_001078952
	dmrt2.L	2a/2b	1L (scaffold106782), 2148326-2148123, -	NP_001089725
	dmrt2.S	2a/2b	1S (scaffold214452), 363479-363682, +	-
	dmrt3.L	3	1L (scaffold106782), 2198684-2198481, -	-
	dmrt3.S	4	1S (scaffold214452), 316209-316412, +	-
	dmrt4.L	4	Scaffold115315, 805901-805698, -	NP_001084923
	dmrt4.S	5	1S (scaffold61609), 3296266-3296469, +	-
	dmrt5.L	5	4L (scaffold53263), 1591875-1591672, -	-
	dmrt5.S	5	4S (scaffold69443), 4588839-4588636, -	NP_001089148
	dmw	1	2L (W), 168707-163748, -	NP_001108314
			allMis0.2/allMis1	
Alligator mississippiensis (Chordata, Vertebrata, Reptilia)	*dmrt1*	1	JH732254, 187057-192844, +	XP_006261327
	dmrt2	2a/2b	JH732254, 341199-341455, +	XP_006261330
	dmrt3	3	JH732254, 294799-295002, +	XP_006261329
	dmrt4	4/5	JH734820, 76191-76394, -	XP_006268224
	dmrt6	6	JH735043, 21327-21530, +	XP_006268809
	dmrt7	7	JH735570, 112895-113098, +	-

Biology Open • Supplementary information

Species	Gene name	Subset	DM domain region (Chr. or scaffold, start-end, strand)	Accession number, gene ID, protein ID
			GRCm38/mm10	
Mus musculus (Chordata, Vertebrata, Mammalia)	*Dmrt1*	1	19, 25506052-25509733, +	NP_056641
	Dmrt2	2a/2b	19, 25673797-25675574, +	NP_665830
	Dmrt3	3	19, 25610862-25611065, +	NP_796334
	Dmrt4	4/5	4, 89688543-89688746, +	NP_783578
	Dmrt5	4/5	4, 109980046-109980249, +	NP_758500
	Dmrt6	6	4, 107683962-107684162, -	NP_063925
	Dmrt7	7	7, 24872584-24872861, +	NP_082008
	Dmrt8.1	8	X, #-#, -	NP_081867
	Dmrt8.2	8	X, #-#, +	NP_001034205
	Dmrt8.3	8	X, #-#, +	NP_001136163
			GRCh37/hg19	
Homo sapiens (Chordata, Vertebrata, Mammalia)	*DMRT1*	1	9, 842043-847013, +	NP_068770
	DMRT2	2a/2b	9, 1051959-1053745, +	NP_006548
	DMRT3	3	9, 977065-977268, +	NP_067063
	DMRT4	4/5	9, 22447333-22447536, +	NP_071443
	DMRT5	4/5	1, 50421147-50421350, -	NP_115486
	DMRT6	6	1, 53459463-53459666, +	NP_149056
	DMRT7	7	19, 41847531-41847817, +	NP_001035373
	DMRT8	8	X, #-#, +	NP_149042

Note. † indicates that DM domain region could not determine because of unknown (N) or unfound sequences in genome. *dmd-3*, *dmd-8*, and *mab-3* have two DM domain regions. * and ** indicate DM domain regions on 5’ and 3’ sides, respectively. # indicates that DM domain region was undetermined because *DMRT8* orthologues have partially the regions.

Biology Open • Supplementary information
